# Barriers decouple population dynamics of riverine fish, and asynchrony of subpopulations promotes stability within fragments

**DOI:** 10.1098/rspb.2025.0429

**Published:** 2025-06-18

**Authors:** Carl Tamario, Petter Tibblin, Anders Forsman

**Affiliations:** ^1^ Department of Wildlife, Fish, and Environmental Studies, Swedish University of Agricultural Sciences, Umeå, Sweden; ^2^ Center for Ecology and Evolution in Microbial Model Systems, EEMiS, Department of Biology and Environmental Science, Linnaeus University, Kalmar, Sweden

**Keywords:** damming, dispersal, metapopulation, persistence, portfolio effect, spatiotemporal

## Abstract

The spatial synchrony framework suggests that asynchrony among subpopulations in different branches of a river network should stabilize the metapopulation. However, how barriers affect this framework remains poorly understood. This is a significant knowledge gap given that population synchrony arises from dispersal and environmental similarity, both of which are influenced by barriers. We empirically evaluated how barriers impact fish population synchrony and, subsequently, the associations between synchrony and metapopulation persistence, productivity, stability and trajectory within fragments. We found that barriers demographically decouple populations by decreasing synchrony in brown trout (*Salmo trutta*) and Eurasian minnow (*Phoxinus phoxinus*), but not in northern pike (*Esox lucius*), suggesting species-specific responses to fragmentation. Additionally, asynchrony had a stabilizing portfolio effect on metapopulation stability at the fragment level that was statistically significant for *S. trutta*. Higher fragment synchrony also made *S. trutta* and *P. phoxinus* populations less stable. The impact of barriers on riverine fish population synchrony emphasizes the need to include barriers in future studies on the causes and consequences of synchrony in rivers. That asynchrony stabilizes populations in some riverine fishes suggests that conservation priorities should lie in restoring or retaining larger fragment sizes and higher branching complexity with intact connectivity.

## 1. Introduction

Understanding how and why different populations co-fluctuate, and how this in turn may impact the persistence of populations, species, communities and ecosystems, are central questions in ecology. Spatial synchrony, which describes the temporal correlation in abundance fluctuations of spatially separated populations, is proposed to mainly arise from dispersal of individuals, by spatially correlated environmental stochasticity (the Moran [1] effect), and by interaction with other trophic levels that are themselves spatially synchronous [2]. Synchrony has implications for the stability of both metapopulations [3] and ecosystems [4], because synchrony among populations has been shown to be directly related to global extinction risk [5]. Asynchrony among populations can thus be viewed as a type of portfolio effect [6]—that is the stabilizing effect through integration of several asynchronous processes that can promote persistence of populations, species and ecosystems [7–10]. However, a recent synthesis suggests that the consequences of dispersal on synchrony, variability and viability of populations are highly context-dependent, and that the grave underrepresentation of empirical evidence calls for further evaluations [11].

A few studies have successfully applied the ecological synchrony framework to understand the spatiotemporal patterns of populations in riverscapes [10,12,13]. A central theme in these studies is that because rivers are structured in a dendritic tree-like network [14], they behave differently from terrestrial or marine systems. Specifically, the branching structure of rivers seems to promote population stability by integrating many asynchronous population trajectories. The interpretation is that populations located in different branches (flow-unconnected [15]) can be asynchronous despite being geographically close since they experience different environments [12], and this asynchrony tends to stabilize populations. While these recent studies have considerably advanced our understanding of fish population dynamics in riverine systems, one unexplored aspect that requires further investigation is how river fragmentation by damming [16,17] impacts population synchrony, and how it can be integrated in this developing framework. We argue that barriers can be viewed as experimental manipulations of the environment that enable efficient evaluation of how fragmentation affects synchrony and long-term persistence of fish populations in riverine systems. A better understanding of these issues is key to the development of better management and protection of biodiversity in these continuously exploited environments.

More than half of large river systems in the world [16,18], and many more small [19], are affected by fragmentation by anthropogenic damming structures, and over one million barriers are estimated to be present in European waterways alone [20]. As one of the most severe and widespread freshwater ecosystem modifiers [21], dams and barriers alter the flux of water, sediment, organisms and energy in all directions, and habitats are degraded, destructed or converted with countless propagating effects on the ecosystem and its inhabitants [17,22,23]. Of primary relevance here is that these structures reduce dispersal opportunities for aquatically restricted organisms, like fish [18]. In addition, dams often create an abrupt change in the environmental gradient [23]. For example, dams can regulate the timing, amplitude or duration of flow by either storing or releasing water [24], as well as alter the temperature regime [25], so that populations downstream and upstream of the dam are exposed to different conditions. Fragmentation by damming should therefore influence population synchrony in rivers, potentially with important consequences for population performance, but this has been overlooked in the synchrony literature. The present study seeks to bridge this knowledge gap.

**Figure 1.**
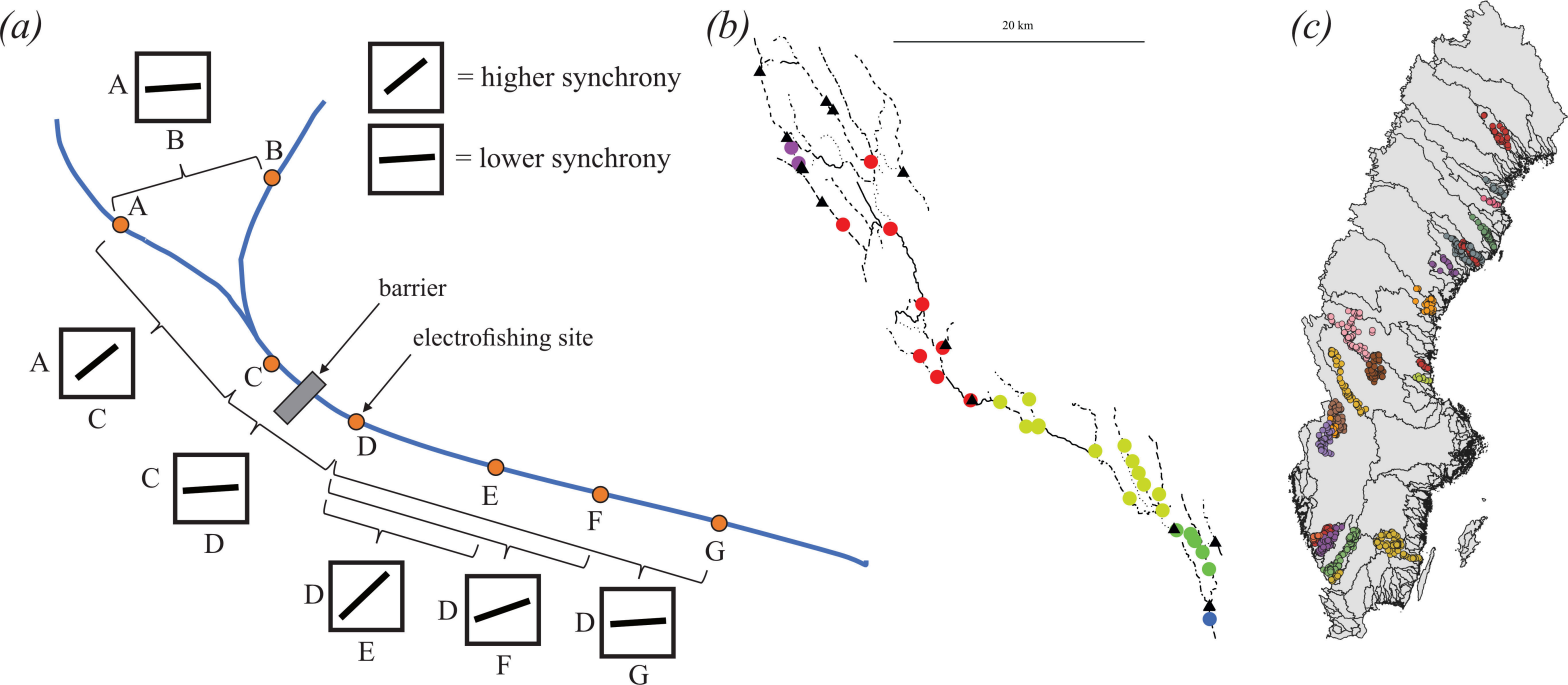
(*a*) Framework for evaluating how spatial synchrony of fish populations in rivers is impacted by fragmentation. Spatial synchrony is the correlation (black squares with correlation indicated by slope of blue line) of spatially separated sampling sites (A to G). Flow-unconnected site-pairs (A ~ B) should have lower synchrony than flow-connected site-pairs (e.g. A ~ C) due to experiencing different environments. Dams should hinder dispersal and create changes in the environment which implies that synchrony of populations in different fragments, i.e. comparisons done over fragment borders (e.g. C ~ D) should be lower than those done within fragments (e.g. A ~ C or D ~ E) at similar distances. Synchrony is generally expected to decrease with watercourse distance due to lower chance of successful dispersal and larger differences in experienced habitat (compare D ~ E, D ~ F and D ~ G). (*b*) One of the catchments (Hörnån river) with round dots representing sampling sites coloured according to fragment identity. Black triangles represent dams that act as barriers fragmenting the river network. (*c*) The spatial distribution of the 927 sampling sites of fish density from 24 catchments (indicated by colour) in the study area of Sweden.

Any desynchronizing effects of barriers should be particularly strong at close distances, where the demographic influence of dispersal between populations should be the strongest, and decay with increasing distance (figure 1). Populations that are flow-unconnected [15] and really distant are likely to fluctuate independently, regardless of whether they are separated by a dam or not. We therefore hypothesized that, at similar pairwise distances, populations separated by barriers will be less synchronous than those that are not separated by barriers, up to a point where their level of synchrony converges (i.e. where stochasticity overrides any such effect) (figure 1). Further, based on the assumption that barriers hinder dispersal to the extent that populations located in different fragments become demographically isolated from each other, we argue that applying the synchrony framework on a per-fragment level offers a powerful approach to evaluate whether and how synchrony affects population persistence, productivity, and stability of fish populations (figure 1). Because synchrony is regularly associated with destabilization of populations [2,4,5,26], we hypothesized that populations in fragments where populations displayed higher synchrony would show larger fluctuations, have lower productivity, and be less persistent. To evaluate this, we extracted and unified spatiotemporal data on abundance of three fish species, dam distribution, and river polylines for 24 catchments in Sweden (figure 1).

## 2. Material and methods

### (a) Study species, data acquisition and river structure

Fish density data were acquired from Swedish Electrofishing RegiStry (SERS) at the Swedish University of Agricultural Sciences (SLU). Electrofishing is a non-lethal fish sampling method mainly conducted in streams where it is possible to wade. Electric current (DC) is used to attract fish to swim towards a handheld anode where they are caught with a dipping net. It is an established and reliable method for quantifying fish density [27] in rivers, see the Swedish and European Standard [28] for detailed descriptions of the method. To evaluate generality and improve inference space, we used three model species*—Salmo trutta* (brown trout), *Phoxinus phoxinus* (Eurasian minnow) and *Esox lucius* (northern pike). These species represent three families of freshwater fish (*Salmonidae*, *Esocidae* and *Cyprinidae*) that are widely distributed in the northern hemisphere. Although coexisting in riverine habitats, these species require different habitats to complete their life cycles and sustain local populations. *S. trutta* is an obligate lotic spawner laying its eggs on gravel [29], *E. lucius* has a strong preference for spawning in lentic habitats with vegetated soft bottoms [30], whereas the *P. phoxinus* is intermediate, preferably spawning on hard bottoms in slow-flowing lotic habitats [31].

Because of the extensive requirement on spatial and temporal coverage of sampling sites to answer our questions, an initial filtering was done where we removed all electrofishing sites with less than six sampling occasions. This allowed us to visually select 24 catchments with sufficient spatial and temporal coverage throughout Sweden (figure 1) with sites located between 56.62 to 66.83 degrees latitude and 12.10 to 21.82 degrees longitude. The temporal span of sampling data was 1951 to 2021, but 90% of the sampling occasions occurred after 1988. Vectored polylines of the rivers in these 24 catchments were downloaded from Lantmäteriet (Land Survey). The spatial distribution of barriers was downloaded from the Swedish Meteorological and Hydrological Institute (SMHI). These three geographic information layers were integrated in the R package *riverdist* [32], which was used to generate tables with the segment and vertex location of all barriers and electrofishing sites. Watercourse routes between all pairwise sites were recorded, and if the route crossed a segment and vertex of a dam, the two sample sites were assigned to different fragments. A matrix was then constructed with this information, which clustered connected sites into fragments. The *riverdist* package also recorded watercourse distance, Euclidean distance and flow-connectivity between all sites. See figure 1b for a visualization of a catchment with sites split into fragments by barriers.

Of the total 1505 dam occurrences within the study area, 224 were classified as removed and were thus filtered away before the spatial data extraction process. The metainformation in the registry for barrier occurrences was very patchy. Of the remaining 1281 dam occurrences, only 301 specify a dam height: the average height of those dam constructions was 6.4 ( ± 19 s.d.) metres. Information on whether barriers had the capacity to regulate the flow, or if they were barriers for fish, was also rarely specified. Rather than designing questions dependent on such patchy data, all remaining barriers were assumed to be barriers for fish.

### (b) Quantifying synchrony

#### (i) Between sites

Synchrony between all sites in each catchment was quantified using Spearman’s correlations with at least six matching pairwise years for each species (*n* = 9998, *n* = 4653 and *n* = 2033 for *S. trutta*, *P. phoxinus* and *E. lucius*, respectively). Correlation analyses have been used to analyse spatial synchrony in several previous studies (Spearman’s correlations [13,26,33] and Pearson’s correlations [10]). We found that site-pairs that did not have any matching years with density greater than 0 led to negatively biased correlations, so to improve data quality, all such site-pairs were excluded.

To evaluate whether the results and conclusions based on estimates of synchrony were robust or influenced to any important degree by the length of the time period, we also performed a sensitivity analysis using only site-pairs with at least 10 matching years (instead of at least 6 matching years). The results were qualitatively similar, as no significance nor direction of effects were altered; these results are available in the electronic supplementary material, but we report the results based on analyses of at least six matching years in the main text below.

Because distances between site-pairs across fragments have the potential to be longer than those within fragments, the distance between-fragment site-pairs was truncated to the maximum distance of site-pairs within fragments (for each catchment, respectively). This resolves the statistical issue of unequal variances (and ranges) between groups, which can be problematic in models with interactions.

Linear mixed models (LMMs) were performed with the R package *lme4* [34]. Because each ‘observation’ in this dataset is based on information obtained from two sites, the data have a ‘pairwise’ correlation structure (one row equals the synchrony, Euclidean distance, watercourse distance, etc. between two sites). Thus, each site (e.g. A, B, C, D) is used for the calculation of multiple synchrony values (e.g. **A**-B, **A**-C, **A**-D, etc.) and the same site sometimes occurs in different columns (e.g. A-**B**, **B**-C). To account for this non-independence issue, we used a multimembership random effect added using *lme4*-wrapper *LmerMultiMember* [35]. With this approach, a synchrony value can be seen as a group with the two sites as ‘members’, and some part of the variation can be partitioned across the sites across groups.

In light of recent studies [13], accounting for dendritic structure is critical in analyses of synchrony in river networks. To evaluate the influence of spatial distribution, flow-connectedness and fragmentation on site-pair synchrony, we constructed an LMM with site-pair synchrony as the response variable, and the main effects of, and the three-way interaction between, ‘water course distance’, ‘flow-connectedness’ and ‘fragment border passing’ as explanatory variables. Random intercept for catchment identity and multimembership effect for site were included as random effects in the models. We analysed the data for each of the three species separately. To ensure that our model maintains the expected Type I error rate under the null hypothesis, we conducted a simulation-based evaluation outlined in the electronic supplementary material. Reassuringly, the proportion of *p*-values below the alpha level (0.05) was approximately 5% across all model terms except the intercept (electronic supplementary material, figure S1), confirming that our approach maintains appropriate Type I error rates.

To improve interpretability of results, we also performed models with flow-unconnected (*n* = 6632, *n* = 2561 and *n* = 1243 for *S. trutta*, *P. phoxinus* and *E. lucius*, respectively) and flow-connected site-pairs (*n* = 3366, *n* = 2092 and *n* = 790 for *S. trutta*, *P. phoxinus* and *E. lucius*, respectively) separately. LMMs with ‘site-pair synchrony’ as response and ‘watercourse distance’, ‘fragment border passing’ and their interaction, as explanatory variables were performed for each species. Random intercept for catchment identity and multimembership effect for site were included as random effects. For flow-connected sites, the interaction effect between ‘watercourse distance’ and ‘fragment border passing’ was not significant (interaction term: *t* = 0.028) for pike, so an additional model with only main effects was performed.

#### (ii) Scaling up site-synchrony to fragment-level

To quantify and compare synchrony at the level of fragments all site-pairs with different fragment identities were filtered away (leaving *n* = 4747, *n* = 2412 and *n* = 730 for *S. trutta*, *P. phoxinus* and *E. lucius*, respectively). The level of synchrony for each fragment (i.e. the metapopulation of the fragment) was then estimated by calculating the arithmetic mean [11,36]. This metric disregards spatial sampling density or dendritic structure so it is likely that fragments that contain sites that are more spaced out, or that are located in different branches, will have lower synchrony values, for example. While being dependent on the spatial sampling distribution, this metric gives an accurate view of the synchrony of the sampled sites.

### (c) Consequences of synchrony for populations

#### (i) The portfolio effect

To quantify the stability (i.e. the portfolio effect response variable; PE) of each fragment metapopulation, we chose the metric and calculated the PE as outlined by the practical guide by Anderson and colleagues [37]. The PE is defined as the ratio of the observed metapopulation CV to the CV of the population as if it were one uniform population [37]. A value of 1.5 in this metric signifies a metapopulation that is 1.5 times more stable than a theoretically uniform population. Noteworthy is that ‘This metric does not address the benefit of increases in portfolio size (e.g. metapopulation size) itself’ [37], implying that unequal sampling in fragments ought not to be a problem.

To choose the appropriate PE metric, we first plotted the mean and variance of each subpopulation time series across all metapopulations on log-log axes, which showed a linear (as opposed to nonlinear) relationship, and retrieved species-specific slopes of z_strutta = 1.61, z_pphoxinus = 1.68, and z_elucius = 1.43. Since all of these *z* are different from *z* = 2 (an assumption of the average-CV PE), we used the more conservative PE metric, the mean-variance CV [37]. To make the estimation of the PE as accurate as possible, the data inclusion criteria were set at similar levels to the salmon example of Anderson et al. [37]: only fragments with at least four subpopulations where each of the subpopulations had at least 10 sampled occasions were included. This resulted in a dataset of *n* = 40 fragments for trout, *n* = 33 fragments for minnow and *n* = 26 fragments for pike. The mean ± s.d. number of sites per fragment was 13.7 ± 11.6 for *S. trutta*, 14.8 ± 12.4 for *P. phoxinus* and 16.6 ± 13.32 for *E. lucius*.

We used LMMs to evaluate the relationship between fragment mean synchrony and the mean-variance portfolio effect response variable. Because some catchments might be represented by several fragments, we added catchment identity as a random effect to the model to account for any residual geographical non-independence.

#### (ii) Other population performance metrics

We also quantified five other population performances; (i) occurrence rate, (ii) mean log-density+1, (iii) standard deviation in log-density, (iv) residual standard deviation (RSD) and (v) population trajectory slope, for all respective sites using all the available data (i.e. the entire time series of each site) (table 1). These metrics thus have a larger sample size than for the portfolio effect above. The first three measurements were calculated at site level and then aggregated to fragment level by averaging the values from all sites with the same fragment identity. The last two measurements (residual standard deviation and population trajectory slope) were calculated through performing a model per fragment: the residual standard deviation is calculated, per fragment, from regressing all containing sites’ time series of log-density on years in interaction with site identity and using the residuals to calculate a population variability (Please see electronic supplementary material, figure S2 for visual explanation). An important advantage of this last way to estimate variability of sites in fragments is that it also detrends the data (i.e. preventing longer-term trends in data from being included in estimations of year-to-year variability). The mean fragment trajectory slope was used to evaluate if level of synchrony is associated with population trajectory. This resulted in a dataset of *n* = 101 fragments for trout, *n* = 70 fragments for minnow, and *n* = 62 fragments for pike. The mean ± s.d. number of sites per fragment was 7.35 ± 9 for *S. trutta*, 8.9 ± 10.2 for *P. phoxinus* and 8.8 ± 10.8 for *E. lucius*.

**Table 1.** Measurements of population performance calculated on fragment-level and their explanations.

response variable	meaning
portfolio effect	portfolio effect, mean-variance CV calculated according to Anderson et al. [37]
occurance rate	mean occurrence based on all sampled occasions
mean density	mean log-density = mean(log(density+1))
standard deviation (s.d)	standard deviation of density time series
residual s.d	standard deviation of residuals from time series models (see electronic supplementary material, figure S2)
population trajectory	beta coefficient (i.e. slope) from time series models (see electronic supplementary material, figure S2)

A justification for using a range of different metrics is that they all capture slightly different ecological aspects of population performance [11]. Some, but not all, of the metrics of population performance were positively correlated, and the pairwise correlations differ somewhat between the three species (electronic supplementary material, figures S3–S5).

To evaluate the association between synchrony and these population performance metrics, we performed LMMs with mean fragment synchrony as explanatory variable, and the five different measures of performance as responses, respectively, for each species. The test statistics were recorded in a table.

**Table 2.** Model output from a LMM evaluating the effects of barriers on synchrony of populations of a) *S. trutta*, b) *P. phoxinus* and **C**) *E. lucius*. Due to a non-significant interaction term between distance and barriers for *E. lucius*, an additional main effect-only model d) (*E. lucius* (2)) was also performed.

	species	term	transformation	estimate	std. error	*t-*value	*p*‐value	*R* ^2^
a.	*S.trutta*	(intercept)		0.396	0.03	13.11	<0.0001	**c**onditional: 0.242
		watercourse distance (dist)	(sqrt)	−0.001	1.32e−04	−11.15	<0.0001	
		fragment crossing (frag)		−0.161	0.034	−4.79	<0.0001	marginal: 0.066
		dist: frag		7.66e−04	2.16e−04	3.55	0.0004	
b.	*P. phoxinus*	(intercept)		0.374	0.027	13.63	<0.0001	conditional: 0.250
		watercourse distance (dist)	(sqrt)	−0.001	1.47e−04	−7.78	<0.0001	
		fragment crossing (frag)		−0.157	0.043	−3.66	0.0003	marginal: 0.039
		dist : frag		0.001	2.82e−04	3.98	<0.0001	
c.	*E. lucius* (1)	(intercept)		0.29	0.045	6.50	<0.0001	conditional: 0.245
		watercourse distance (dist)	(sqrt)	−1.13e−04	2.98e−04	−0.38	0.704	
		fragment crossing (frag)		0.027	0.051	0.54	0.590	marginal: 0.002
		dist: frag		−1.64e−04	3.94e−04	−0.42	0.677	
d.	*E. lucius* (2)	(intercept)		0.297	0.042	7.13	<0.0001	conditional: 0.245
	(*no interaction*)	watercourse distance (dist)	(sqrt)	−1.88e−04	2.40e−04	−0.78	0.433	
		fragment crossing (frag)		0.01	0.028	0.35	0.725	marginal: 0.001

The Benjamini–Hochberg method was used to adjust the *p*-values for all population metrics, including the portfolio effect in the previous section.

### (d) Does adding information on barriers improve a previous framework?

To investigate if adding barriers adds significantly to the predictive power of the recent development of a ‘geography of spatial synchrony in dendritic river networks’ [13], we replicated the fluvial synchrogram variables defined therein in our dataset. The synchrograms developed by Larsen and colleagues in 2021 [13] offer insights into the effects of both hydrological connectivity and upstream dependence among populations, while also capturing relationships among populations across adjacent tributaries and the broader landscape context. They calculate Euclidean distance (d_E_) and watercourse distance (d_W_) and their quotient (d_E_/d_W_) between all pairwise sites and subsequently recognize that sites can be separated by four functionally different distances: D1, D2, D3 and D4. The authors present four major expectations based on these four combinations of pairwise distances:

— D1: populations with small and similar d_E_ and d_W_ values are likely on the same network branch, displaying high synchrony. They are close both in terms of physical distance and water flow, suggesting a strong potential for interaction and shared dynamics.
— D2: populations with large and equal d_E_ and d_W_ values are likely located on the same branch but are more distant. They are still expected to show intermediate synchrony, influenced by a combination of dispersal and the Moran effect.
— D3: populations with small d_E_ but much larger d_W_ values are likely situated in separate but nearby branches. They should display intermediate synchrony, primarily driven by a Moran effect. This suggests that even though they may not be directly connected by water flow, there might still be shared environmental influences.
— D4: populations with large d_E_ and much larger d_W_ values are positioned on distant and separate branches. They are expected to show the lowest degree of synchrony due to their significant spatial and hydrological separation.

We set out to explore whether and how the inclusion of barriers enhances the predictive capacity of this framework within our dataset. Euclidean distance, watercourse distance and their quotient (d_E_/d_W_) were calculated for all sites. The classification was then done river-catchment-wise, so that the delineation into short or long d_E_, and high or low d_E_/d_W_, was set at the catchment-wise median values, and all pairwise observations in the present study were subsequently categorized into the four classes of distance (D1, D2, D3, D4). Two models were then performed per species, one that modeled the synchrony main effect of the synchrogram category, and another model that included main effect of synchrogram and its interaction effect with fragment border crossing (1/0). The Akaike information criterion (AIC) was then used to compare the parsimony of these two (per species) models [38]. We hypothesized that closely located sites (those separated by D1) would show the largest divergence in synchrony when either separated or not separated by barriers. Because of the nature of synchrony decay with distance, we hypothesized that differences between models would be smaller for D2 through D4.

### (e) Softwares and packages used

All data manipulations, calculations and analyses were done in R language [39]. Widely used packages include those in the *tidyverse* ecosystem [40], *lme4* [34], *PerformanceAnalytics* [41] and *riverdist* [32]. We used the R package *car* [42] with the *Anova* function to estimate *p*-values. We used the R package *performance* [43] with the *r2* function to estimate conditional and marginal *R*^2^ values. Correction for multiple testing was done using Benjamini–Hochberg with the *p.adjust* function from *stats* [39] package.

## 3. Results

### (a) Barriers disrupt subpopulation synchrony

**Figure 2.**
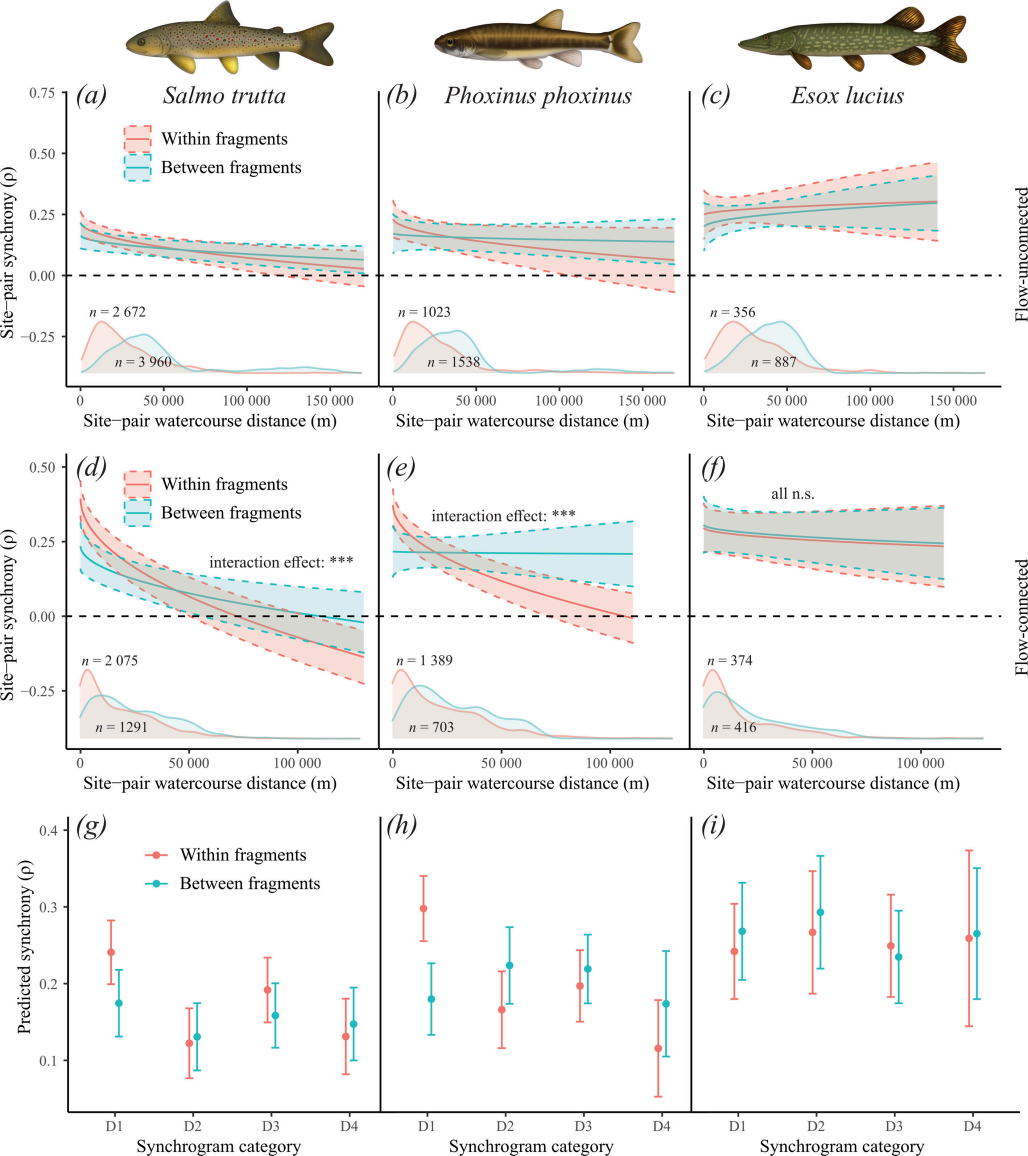
Estimated marginal mean effects from LMMs of water course distance and fragment border crossing (0/1), and their interaction, on site-pair synchrony of three freshwater fish species in Sweden for (*a*–*c*) flow-unconnected sites (see electronic supplementary material, table S1) and (*d*–*f*) flow-connected sites (see table 2). While barriers did not contribute to decoupling subpopulations that were flow-unconnected (*a–c*), barriers did decouple flow-connected subpopulations of (*d*) *S. trutta* and (*e*) *P. phoxinus* but not (*f*) *E. lucius*. The density distributions in the lower part of panels (*a–f*) represent data coverage and the two *n*-values in the lower part of the panels indicate the sample sizes of synchrony values within (upper *n*) and between (lower *n*) fragments, respectively. The *x*-axis is back-transformed from square-root space. Asterisks denote the level of statistical significance. The re-analysis of the fluvial synchrogram [13] showed that predictions were significantly altered and improved (according to AIC) by adding information on barriers for (*g*) *S.trutta*, (*h*) *P. phoxinus* but not for (*i*) *E. lucius*. Synchrony was higher between geographically proximate populations (D1) of *S. trutta* and *P. phoxinus* when belonging to the same, rather than different, fragments.

The first set of LMMs showed that that there was a significant three-way interaction between flow-connectedness, barriers, and distance on the synchrony of populations of *S. trutta* (effect of three-way interaction, *p* = 0.03, conditional *R*^2^ = 0.162, marginal *R*^2^ = 0.032), but not for *P. phoxinus* (effect of three-way interaction, *p* = 0.09, conditional *R*^2^ = 0.185, marginal *R*^2^ = 0.033) and *E. lucius* (effect of three-way interaction, *p* = 0.98, conditional *R*^2^ = 0.218, marginal *R*^2^ = 0.002) (electronic supplementary material, table S1). For trout, the synchrony of site-pairs belonging to different water flows (i.e. flow-unconnected) was akin to that of site-pairs belonging to different fragments (i.e. separated by barriers) (illustrated in figure 2*a*–*c*; electronic supplementary material, table S1), suggesting that barriers increase asynchrony between populations similar to if the populations were in different river branches. To facilitate statistical interpretation of the effect of barriers on synchrony, we excluded all flow-unconnected site-pairs and ran a simpler model (table 2). The results showed that populations fluctuate more independently if separated by barriers in *S. trutta* (effect of interaction between site-pair distance and barrier, *p* < 0.0001, conditional *R*^2^ = 0.242, marginal *R*^2^ = 0.066) and *P. phoxinus* (effect of interaction between site-pair distance and barrier, *p* < 0.0001, conditional *R*^2^ = 0.250, marginal *R*^2^ = 0.039), but not in *E. lucius* (effect of interaction between site-pair distance and barrier, *p* = 0.67, conditional *R*^2^ = 0.245, marginal *R*^2^ = 0.002), compared to if they were unseparated by barriers (figure 2*d*–*f*; table 2). After removing the interaction effect for pike, not even distance or barriers influenced synchrony (table 2d). The synchrony for *S. trutta* and *P. phoxinus* was 67% (0.40 versus 0.24) and 68% (0.37 versus 0.22) higher at the intercept for site-pairs unseparated by barriers compared to site-pairs separated by barriers. For site-pairs unseparated by barriers, synchrony was initially high and subsequently declined with site-pair distance. At distances greater than 20−25 km (on average) the synchrony between unseparated and separated site-pairs converged. The sensitivity analysis showed that the results were qualitatively similar when synchrony values were only calculated for ≥10 years (as opposed to ≥6 years) as no significance nor direction of effects were altered (electronic supplementary material, table S2).

### (b) Fragment asynchrony may increase population stability

**Figure 3.**
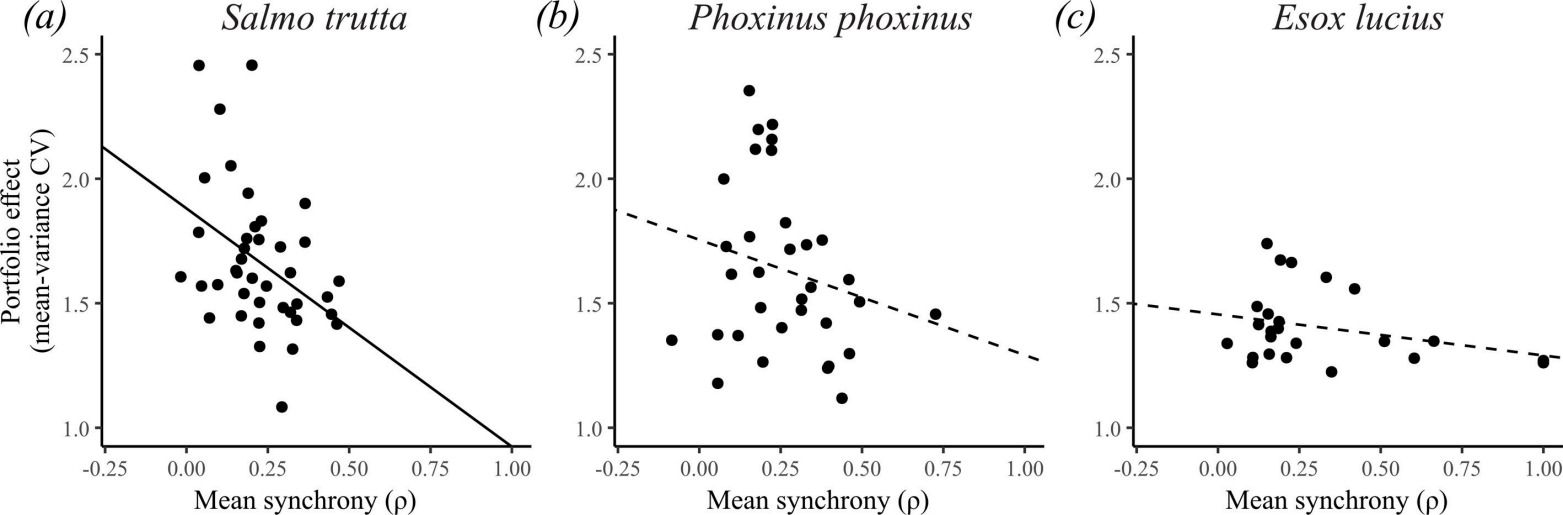
Associations between mean fragment synchrony and the portfolio effect in (*a*) *S. trutta*, (*b*) *P. phoxinus* and (*c*) *E. lucius*. Full or dashed lines indicate the significance of the slope.

In this analysis, we evaluated the consequences of synchrony for different metrics of population performance within fragments, using them as in-site experimental units. The results showed a significant negative relationship between arithmetic mean fragment synchrony and the mean-variance CV of population size in *S. trutta* (LMM, *p_adj_* = 0.02, conditional *R*^2^ = 0.26, marginal *R*^2^ = 0.16, *n* = 40), indicating a stabilizing portfolio effect of fragment asynchrony on population dynamics. The two other species also showed negative albeit non-significant relationships (LMMs; *P. phoxinus: p* = 0.20, conditional *R*^2^ = 0.12, marginal *R*^2^ = 0.05, *n* = 33; *E. lucius*, *p* = 0.14, conditional *R*^2^ = 0.10, marginal *R*^2^ = 0.09, *n* = 26); the higher *p*-values for these species might partly be due to lower sample sizes compared to *S. trutta* (figure 3). Additionally, there were significant and positive associations between fragment mean synchrony and standard deviation in log-density for *S. trutta* (LMM, *p_adj_*<0.0001, conditional *R*^2^ = 0.36, marginal *R*^2^ = 0.14, *n* = 101) and *P. phoxinus* (LMM, *p_adj_* = 0.02, conditional *R*^2^ = 0.45, marginal *R*^2^ = 0.11, *n* = 70), and residual s.d. for *P. phoxinus* (LMM, *p_adj_* = 0.02, conditional *R*^2^ = 0.39, marginal *R*^2^ = 0.11, *n* = 70), indicating that synchrony destabilizes population fluctuations. There were no significant associations between fragment mean synchrony and the other population performance metrics (occurrence rate, mean density and population trajectory; table 3; scatterplots are illustrated in electronic supplementary material, figure S6).

**Table 3.** Matrix with *t*-values from LMMs evaluating the effects of synchrony on five measurements of population performance (see table 1 for explanations on responses). Nominal *p*-values are noted in parentheses, and adjusted *p*-values by the Benjamini–Hochberg method are noted in square brackets. Significance after correction is indicated by bold face. Singular models are noted with †.

population performance metric	species		
	*S. trutta*	*P. phoxinus*	*E. lucius*
portfolio effect	**−2.76(0.006)[0.026]**	−1.28(0.20)[0.310]	−1.477(0.14)[0.279]
occurrence rate	−0.62†(0.53)[0.6]	1.17(0.24)[0.33]	−0.6† (0.55)[0.6]
mean log-density	−1.08(0.28)[0.36]	1.81(0.07)[0.19]	−2.03(0.04)[0.15]
standard deviation (s.d.)	**4.3( <0.0001)[ <0.0001]**	**2.99(0.003)[0.02]**	−1.27(0.2)[0.31]
residual s.d.	1.79(0.07)[0.19]	**2.9(0.004)[0.02]**	−1.26(0.21)[0.31]
population trajectory	1.66^†^ (0.1)[0.22]	−0.24(0.81)[0.81]	−0.58(0.56)[0.6]

### (c) Information on barriers improves parsimony of a previous framework

The results show that the model which includes information on which subpopulations are separated by barriers performed significantly better than the model without barriers for *S. trutta* (AIC = 7375.0 versus 7400.2; *p* = 1.1 × 10^−6^) and for *P. phoxinus* (AIC = 3172.5 versus 3221.1; *p* = 1.48 × 10^−11^), but not for *E. lucius* (AIC = 1041.5 versus 1035.5; *p* = 0.72). The largest difference in prediction for the *S. trutta* and *P. phoxinus* synchrogram was seen between closely located sites (D1) for which synchrony was vastly lower when separated by barriers (figure 2*g*–*i*), which is in line with our hypothesis.

## 4. Discussion

Overall, the patterns of spatial synchrony of riverine fish populations that emerge in this study largely agree with predictions from theory and previous studies. Our results show that populations of some fish species display spatial structuring along rivers, with close populations fluctuating more similarly than distant populations, and populations in different river branches (i.e. flow-unconnected site-pairs) fluctuating more independently than those within the same branch. The results also show that populations of some fish species fluctuate much more independently when separated by barriers, indicating that barriers cause abrupt discontinuations in the populations’ gradual nature, particularly in *S. trutta*. This is expected because barriers may both decrease dispersal opportunities [18,44] and alter the habitat [23,24,45], two central drivers of spatial synchrony in populations. For both *S. trutta* and *P. phoxinus*, at the theoretical site-pair distance of zero (i.e. at the intercept), synchrony was around two-thirds higher (67% and 68%) if populations were connected compared to if they were separated by barriers. As expected, with increasing pairwise distance, the synchrony in unseparated versus barrier-separated subpopulations eventually evened out. That the desynchronizing effects of barriers were strongest at short site-pair distances was emphasized by the results from the re-analysis of the framework of Larsen and colleagues [13]. Synchrony of site-pairs separated by synchrogram category D1 (i.e. close distance, and close d_E_/d_W_, that is with high functional connectivity) was higher when both sites were within a fragment than if they belonged to different fragments (for *S. trutta* and *P. phoxinus*). *E. lucius* displayed synchrony too, but unlike in the other two species, the level of synchrony was not associated with site-pair distance, the presence of barriers, nor with flow-connectivity. Although the sample size for *E. lucius* was smaller than for the other two species, it is unlikely that smaller confidence intervals alone would alter the conclusions for *E. lucius*.

Our findings support that the effects of fragmentation [46] and the level of synchrony [33] are likely to differ between species, likely depending on their life histories and ecological requirements. Earlier studies on spatial synchrony among fish species have related higher levels of synchrony to species having ‘greater dispersal abilities, lower thermal tolerance, and opportunistic strategy’ [33] and ‘synchronized reproduction during the wet season, high fecundity, small egg size and high gonado-somatic index’ [47]. Our findings further suggest that the responses to synchronizing drivers are differently modified by spatial scale in different species. Populations of *S. trutta* and *P. phoxinus* were sensitive to synchronizing effects at short distances and less sensitive to synchronization over long distances. This indicates that the processes driving synchrony in these two species are relatively small-scale (spanning only tens of kilometers), such as dispersal or local environmental conditions. *S. trutta* is a salmonid, generally regarded as having high dispersal capacities [48]. *P. phoxinus*, despite their small body size, are also capable of partaking in migrations [49] and, in many cases, show similar patterns to those of *S. trutta* in the present study. In contrast to the two former species, *E. lucius* seems to be sensitive to synchronizing effects over both short and long distances, since they display synchrony regardless of site-pair distance and across fragments. Although *E. lucius* has migrating forms [50], it is a relatively sedentary species that inhabits lakes, wetlands/pools, and backwaters in rivers [51]. The synchronous population fluctuations in *E. lucius* might therefore be affected by environmental variables that vary more or less uniformly over entire catchments, like warm or cold years. Together, these results and reasoning are in agreement with a previous evaluation of climatic versus dispersal drivers of synchrony [52], in which the authors suggested that synchrony within lakes (being analogous to fragments) was driven by dispersal, while synchrony across lakes was driven by climate.

The desynchronizing effects of barriers demonstrated by our results were not visible when subpopulations were located in different river branches. This might be because the previously identified [10,12,13] synchrony-buffering effect of river branching might have already decoupled populations to such an extent that any further potential desynchronizing effect of barriers is masked. We would much like to emphasize that asynchrony induced by branching complexity should *not* be equated with the asynchrony induced by fragmentation, and their potential effects on population stability should not be expected to be similar. The crucial difference is that dispersal, and therefore dispersal-induced stability, is possible between asynchronous tributaries, but impossible or highly restricted between asynchronous fragments separated by barriers. Additionally, dams restrict access to important spawning, rearing and foraging habitats, and so have negative effects on other aspects of population dynamics (e.g. their abundance) than asynchrony [53].

A major challenge in the spatial synchrony research field is to discriminate the effects of dispersal from those of regional stochasticity or other synchronizing factors [2]. In most systems, these co-occur [2], which complicates identifying the cause(s) of synchrony. Dams or other barriers can prevent dispersal in one or both directions and alter the habitat, or both, or none, depending on their construction [23]. The difference between these different types of dams could assist in future attempts to disentangle alternative synchronizing drivers. However, such an evaluation requires carefully collected and detailed data for each individual dam, which we did not have for the present study.

We hypothesized that synchrony would impact fish population performance on a per-fragment basis. The results suggested that greater asynchrony at the level of fragments tended to dampen between-year fluctuations in population size within fragments in all three species, in agreement with expectations from theory, but with the stabilizing portfolio effect being significant only for *S trutta.* The results for two of the other investigated metrics of population performance (s.d. and residual s.d.) also indicated that higher fragment asynchrony was associated with dampened density fluctuations, but not with occurrence rate, mean density or population trajectory. That asynchrony has potential to promote the stability of populations has been shown theoretically [5,12] and empirically [10,13], and at different hierarchical levels of biological diversity [4].

However, previous empirical investigations into the links between dispersal, synchrony and population have arrived at mixed results and conclusions. Thus, a recent meta-analysis [11] of experimental manipulation studies comprising a range of aquatic and terrestrial organisms suggest that dispersal increases population synchrony but has only weak effects on population variability. However, both the sign and strength of the dispersal effects on population synchrony and variability were highly context dependent, being modulated by taxa (as in our study), as well as by environmental heterogeneity, type of perturbations and particularly sensitive to study duration, suggesting that effects of dispersal are not generalizable across systems. Comparisons of results and conclusions are complicated further by that Yang and colleagues [11] quantified consequences of dispersal, but their analyses did not directly evaluate the effects that synchrony may have on population performance. Their meta-analysis also did not include any studies of fish or other organisms in dendritic systems, which adds to the value of the present study.

To conclude, our results support that barriers contribute to a demographical isolation of populations of *S. trutta* and *P. phoxinus*, as subpopulations that belonged to different fragments fluctuated much more independently than those separated by similar distances but belonging to the same fragment. That this was not the case in *E. lucius* shows that consequences of dispersal barriers and drivers of synchrony patterns were species-specific. That the predictions from the synchrogram [13] became more accurate when adding information on barriers further emphasizes the need to include dispersal barriers in future empirical evaluations and theoretical developments of the causes and consequences of spatial synchrony of fish population dynamics in river networks. The synchrony-buffering aspect of branching previously demonstrated [12,13] was also evident in our study system; when subpopulations were not flow-connected, the presence of barriers did not have any additional effect on subpopulation synchrony. Lastly, our present results support the idea that asynchrony of subpopulations within fragments may contribute with a stabilizing portfolio effect. Together, this has important implications for fragmented riverine ecosystems. Conservation and restoration priorities should lie in promoting processes that can contribute to higher asynchrony but with preserved connectivity, such as restoring or retaining larger fragment sizes and higher branching complexity.

## Data Availability

The datasets generated and analysed during the current study are available from the Dryad Digital Repository [54]. Supplementary material is available online [55].
